# CCH-Pd complex anchored on ZrFe_2_O_4_ nanoparticles as a novel magnetic catalytic for C-C coupling reactions

**DOI:** 10.1016/j.heliyon.2024.e37683

**Published:** 2024-09-19

**Authors:** Zhino Mohammed Sdiq, Hadi Pourmokhtar, Fatemeh Keshavarzi

**Affiliations:** a Bright Technical and Vocational Institute Sulaymaniyah, Iraq; b Faculty of Chemistry, University of Tabriz, Iran; c Student Research Committee, School of Public Health, Kermanshah University of Medical Sciences, Kermanshah, Iran

**Keywords:** Cinchonine, Suzuki, ZrFe_2_O_4_, Pd

## Abstract

In the present work, Palladium nanoparticles were supported on Cinchonine functionalized ZrFe_2_O_4_ MNPs. The structure, morphology, and physicochemical properties of the particles were characterized through different analytical techniques, including fourier transformed infrared spectroscopy (FT-IR), scanning electron microscopy (SEM), transmission electron microscopy (TEM), inductively coupled plasma (ICP), X-ray powder diffraction (XRD), thermogravimetric analysis (TGA), energy dispersive X-ray spectroscopy (EDS) and vibrating sample magnetometer (VSM) techniques. Moreover, it was obtained as an efficient catalyst for Hiyama and Suzuki reactions under green conditions with good to excellent yields. This method has the advantages of simple methodology, high yields, easy work-up, greener conditions, and short reaction times. This catalyst was reused four times in C-C coupling reaction without loss of its catalytic activity. The reused catalyst was characterized by ICP and FT-IR techniques. Heterogeneity and stability of ZrFe_2_O_4_@SiO_2_-S-CCH-Pd were studied by hot filtration test and ICP analysis.

## 1 Introduction

In the past decade, magnetic nanoparticles have been considered for surface functionalization, such as a coating which could result in obtaining significant properties such as photocatalytic activity [1,2]. Although there are many kinds of materials available for coatings of the magnetic nanoparticles, such as metal oxide, noble metals, and polymer materials, silica is still considered to be the best candidate for surface functionalization because it is highly stable against degradation. Nanosized spinel-ferrites have numerous uses including electronic, magnetic, energy, and catalytic applications [3,4]. A wide range of magnetic nanoparticles with easy recycling and separation capabilities have been developed and used as catalysts for the synthesis of various organic compounds. Immobilization of homogeneous catalysts on solid supports facilitates the separation and reuse of expensive noble metal catalysts [[5], [6], [7]]. However, some heterogeneous palladium catalysts show lower reactivity than homogeneous ones possibly due to the leaching of palladium from the supports. In fact, the leached palladium species is responsible for the catalytic activity in most of the cases. Thus, there is a need to design new heterogeneous catalysts that can retain the activities and selectivities of homogeneous catalysts. Now it is well known that the nature of supports plays a key role in palladium-based heterogeneous catalysis. Among different supports, Zirconium ferrite remains the most popular choice because of its relatively low cost, high thermal, and mechanical stability, and good catalytic performance [[8], [9], [10], [11]].

One of the most valuable synthetic approaches to preparing symmetric and asymmetric biaryls is utilizing the palladium-catalyzed Suzuki coupling reaction of aryl boronic acids with aryl halides. This reaction has been extensively employed in the synthesis of polymers, ligands, pharmaceuticals, and advanced materials. Organic transformations catalyzed by transition metals have gained widespread attention over the years as they yield valuable products [[12], [13], [14]]. Palladium-catalyzed Suzuki cross-coupling reaction for the formation of the C–C bond is one such commercially important reaction [15,16]. Despite their importance, most of these palladium-catalyzed reactions suffer from inherent problems related to the separation, instability of the homogeneous catalysts at high temperatures, recovery, Palladium metal aggregation, and precipitation, using expensive ligands and toxic organic solvents [17]. In the past two decades, advances have been made to develop even more efficient catalytic systems for the C-C coupling reaction [18]. Unfortunately, most of these well-known systems, in addition to moderate yields and poor substrate generality have serious disadvantages such as using high reaction temperatures, harmful organic solvents, and excess stoichiometric amounts of palladium promoter [19,20].

In this study, a novel Pd complex supported on ZrFe_2_O_4_ MNPs (ZrFe_2_O_4_@SiO_2_-S-CCH-Pd) was introduced as a new and efficient reusable catalyst for Hiyama and Suzuki reaction under green reaction conditions.

## 2 Experimental

### 2.1 Preparation of ZrFe_2_O_4_@SiO_2_-S-CCH-Pd

**Scheme 1:**
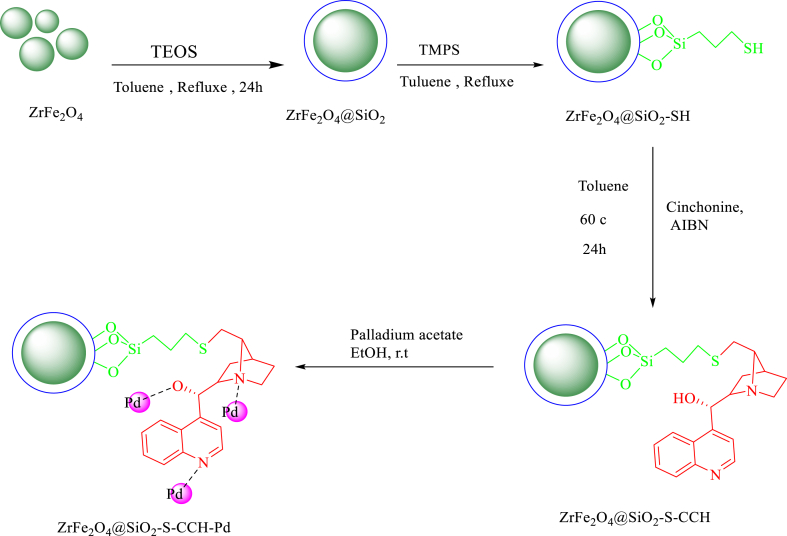
Synthesis of ZrFe_2_O_4_@SiO_2_-S-CCH-Pd.

First, for the synthesis of ZrFe_2_O_4_@SiO_2_ was synthesized according to the previous report [21]. In the next step, ZrFe_2_O_4_@SiO_2_ (1 g) was added to the solution of (3-mercaptopropyl) trimethoxysilane (10 mmol) in toluene (20 mL) and the resultant mixture was under reflux for 22 h. The product was washed with toluene to remove no reacted species and dried at 50 °C for 6 h. A mixture of cinchonine (CCH) (2 mmol), nano ZrFe_2_O_4_@SiO_2_-SH (1 g), and azobisisobutyronitrile (AIBN) (0.03 mmol) in 40 mL of toluene was stirred at 60 °C for 24 h. After removal of the solvent under vacuum, the residue was washed several times with hexane and dried in an oven at 50 °C for 4 h. The white product, nano ZrFe_2_O_4_@SiO_2_-S-CCH obtained, was used for the subsequent reaction without further purification. In continues, 1.0 g of ZrFe_2_O_4_@SiO_2_-S-CCH was added to 50 mL ethanol solution of palladium acetate (2.5 mmol), and the mixture was refluxed for 24 h. The Final product was collected by separation magnetically, copiously washed with ethanol to remove any loose metal species, and dried under vacuum to give the magnetic ZrFe_2_O_4_@SiO_2_-S-CCH-Pd catalyst (Scheme 1).

### 2.2 Preparation of Suzuki reaction

**Scheme 2:**
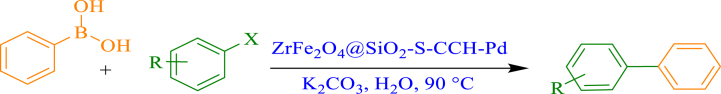
ZrFe_2_O_4_@SiO_2_-S-CCH-Pd catalyzed Suzuki coupling reactions.

A mixture of aryl halides (1 mmol), phenylboronic acid (1.1 mmol), Potassium Carbonate (1.5 mmol), H_2_O (2 mL), and the ZrFe_2_O_4_@SiO_2_-S-CCH-Pd (0.02 g) complex was stirred under air at 90 °C. Next, the progress of the reaction was checked using TLC. The mixture was cooled and filtered. The extracts were washed with EtOAc and dried over Na_2_SO_4_ (1.5 g). After the removal of the solvent, the desired products were obtained with excellent yields (Scheme 2).

### 2.3 Preparation of Hiyama reaction

**Scheme 3:**
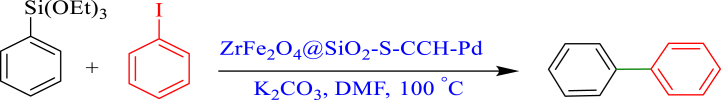
ZrFe_2_O_4_@SiO_2_-S-CCH-Pd catalyzed Hiyama coupling reactions.

A mixture of aryl halides (1 mmol), Triethoxyphenylsilane (1.2 mmol), Potassium Carbonate (1.5 mmol), DMF (2 mL), and the ZrFe_2_O_4_@SiO_2_-S-CCH-Pd (0.01 g) complex was stirred under air at 100 °C. The progress of the reaction was checked using TLC. Next, the mixture was cooled and filtered. The extracts were washed with EtOAc and dried over Na_2_SO_4_ (1.5 g). After the removal of the solvent, the desired products were obtained with excellent yields (Scheme 3).

### 2.4 Selected NMR data

**3-Nitro-1,1′-biphenyl:**^1^H NMR (400 MHz, DMSO)**:** *δ*_H_ = 7.86–8.06 (m, 9H) ppm.

**1,1′-Biphenyl:**^1^H NMR (400 MHz, DMSO)**:** *δ*_H_ = 7.15–7.44 (m, 10H) ppm.

**2-MeO-1,1′-biphenyl:**^1^H NMR (400 MHz, DMSO)**:** *δ*_H_ = 7.25–7.47 (m, 9H), 4.09 (m, 3H) ppm.

**4-Nitro-1,1′-biphenyl:**^1^H NMR (400 MHz, DMSO)**:** *δ*_H_ = 7.30–7.56 (m, 9H) ppm.

**[1,1′-Biphenyl]-4-amine:**^1^H NMR (400 MHz, DMSO)**:** *δ*_H_ = 7.34–7.67 (m, 9H), 4.91 (s, 2H) ppm.

#### 2.4.1 Catalyst characterizations

**Fig. 1:**
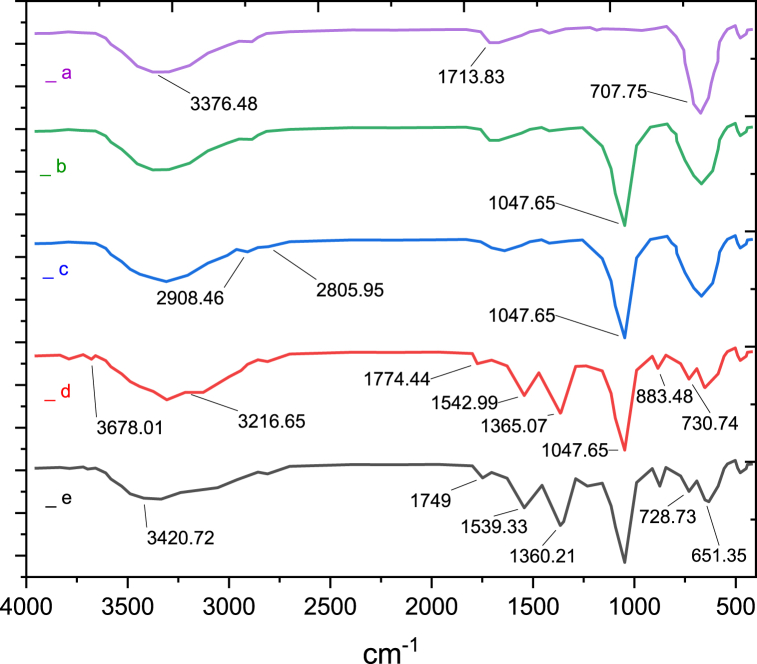
Comparative study of FT-IR spectra of a) ZrFe_2_O_4_, b) ZrFe_2_O_4_@SiO_2_, c) ZrFe_2_O_4_@SiO_2_-SH, d) ZrFe_2_O_4_@SiO_2_-S-CCH, e) ZrFe_2_O_4_@SiO_2_-S-CCH-Pd.

To ensure the successful functionalization of ZrFe_2_O_4_@SiO_2_-S-CCH-Pd MNPs, FT-IR spectroscopy was carried out with KBr pellet (Fig. 1). The absorption bands for ZrFe_2_O_4_ (Fig. 1a), and ZrFe_2_O_4_@SiO_2_ (Fig. 1b) were consistent with our previous reports respectively. In Fig. 1c, the presence of peaks at 2805, and 2908 cm^−1^ in ZrFe_2_O_4_@SiO_2_-SH have belonged to C–H stretching vibrations. In Fig. 1d, the characteristic bands at 1365, 1542, 3216, and 3678 cm^−1^ were related to the presence of ZrFe_2_O_4_@SiO_2_-S-CCH. The presence of absorption bands at 1365 cm^−1^ can correspond to carbon–nitrogen stretching vibrations, 3216 cm^−1^ and 3678 cm^−1^ are associated with the OH and NH vibrations, which confirm the successful attach. Finally, the change in the intensity of the peaks of ZrFe_2_O_4_@SiO_2_-S-CCH-Pd confirms the coordination of the nitrogen atom of the amino groups to Pd (Fig. 1e).

**Fig. 2:**
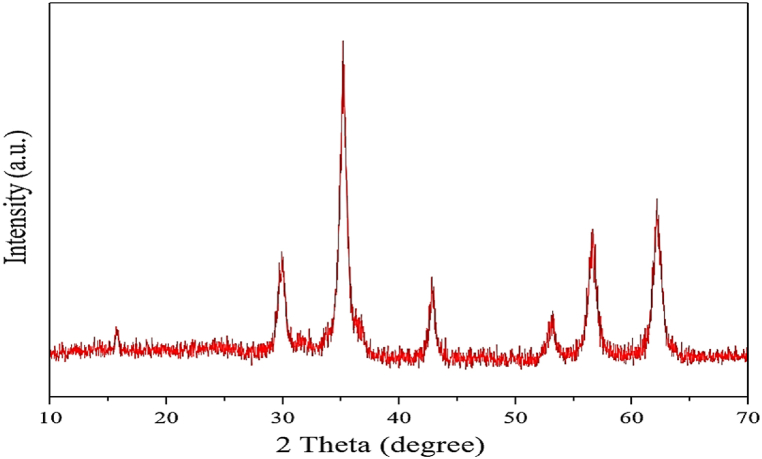
XRD spectrum of ZrFe_2_O_4_@SiO_2_-S-CCH-Pd

The X-ray diffraction patterns of ZrFe_2_O_4_@SiO_2_-S-CCH-Pd complex nanoparticles are shown in Fig. 2. The crystalline peaks at diffraction angles 2θ = 29.01, 36.44, 43.34, 53.52, 57.87, and 62.11 correspond to the (2 2 0), (3 1 1), (4 0 0), (4 2 2), (5 1 1) and (4 4 0) reflections, respectively, which are in agreement with the standard patterns of inverse cubic spinel magnetite crystal structure. According to this analysis, it was confirmed that modifying the surface and performance of ZrFe_2_O_4_ MNPs did not lead to its phase change (Fig. 2).

**Fig. 3:**
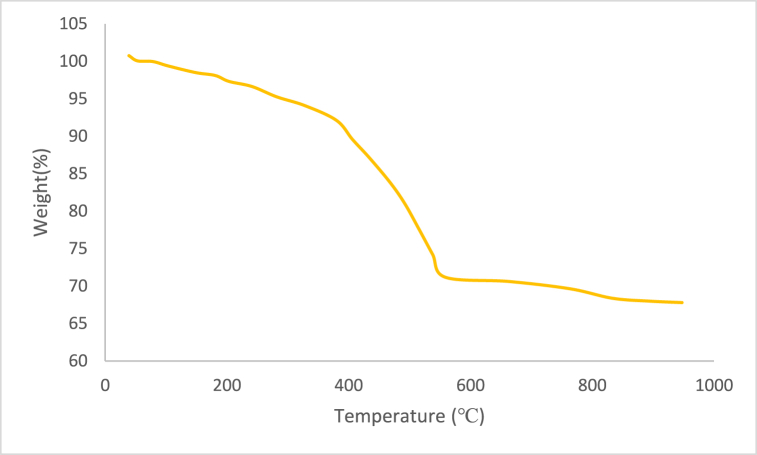
TGA curve of ZrFe_2_O_4_@SiO_2_-S-CCH-Pd.

The thermal stability of catalyst was investigated using TGA (Fig. 3). In the TGA curve of the ZrFe_2_O_4_@SiO_2_-S-CCH-Pd, the first weight loss stage (below 250 °C) can be ascribed to the evaporation of physically adsorbed water molecules in the composition. The next weight loss stage (250–550 °C) can be ascribed to the decomposition of the SiO_2_-S-CCH-Pd organometallic complex on the surface of ZrFe_2_O_4_ MNPs. Also, it should be noted that the ZrFe_2_O_4_@SiO_2_-S-CCH-Pd can stable without any decomposition and it can be used in catalytic reactions of temperature up to 350 ^°^C.

**Fig. 4:**
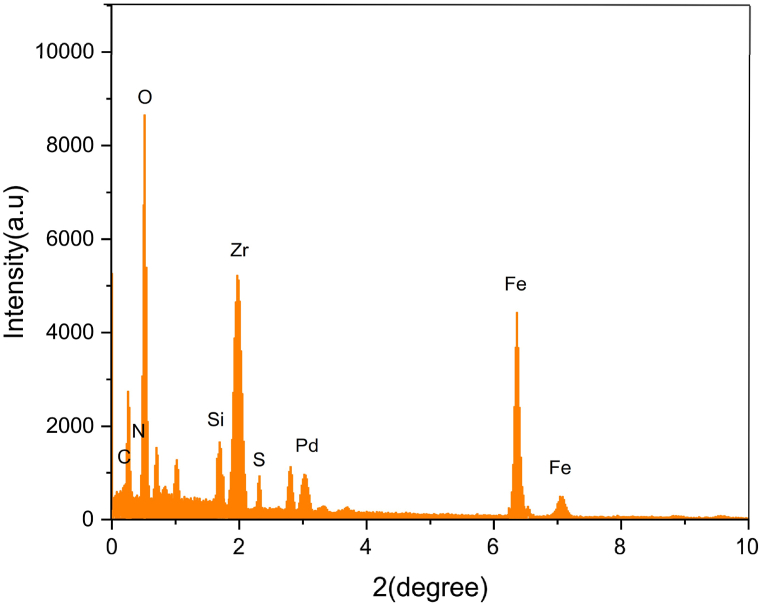
EDS analysis of ZrFe_2_O_4_@SiO_2_-S-CCH-Pd.

The presence of elements in ZrFe_2_O_4_@SiO_2_-S-CCH-Pd was studied by EDX analysis. As shown in Fig. 4, confirmed the presence of S, Fe, Si, Zr, N, O, Pd, and C elements in the catalyst according to the database of the EDX pattern (Fig. 4).

**Fig. 5:**
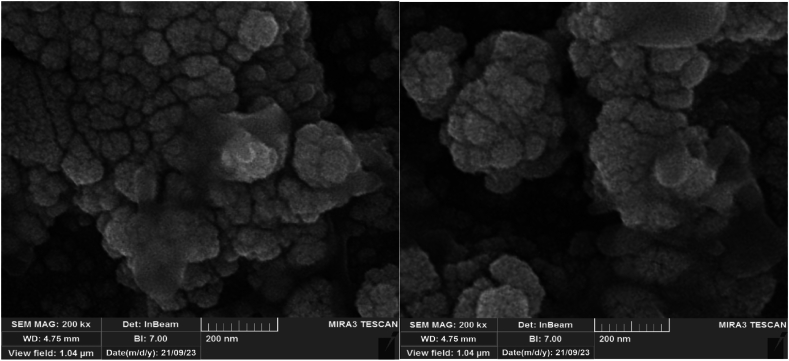
SEM images of ZrFe_2_O_4_@SiO_2_-S-CCH-Pd.

Next, the morphology of ZrFe_2_O_4_@SiO_2_-S-CCH-Pd was studied by SEM (Fig. 5). Also, as it is clear from the pictures, the synthesized nanoparticles are formed in the form of fine grains.

**Fig. 6:**
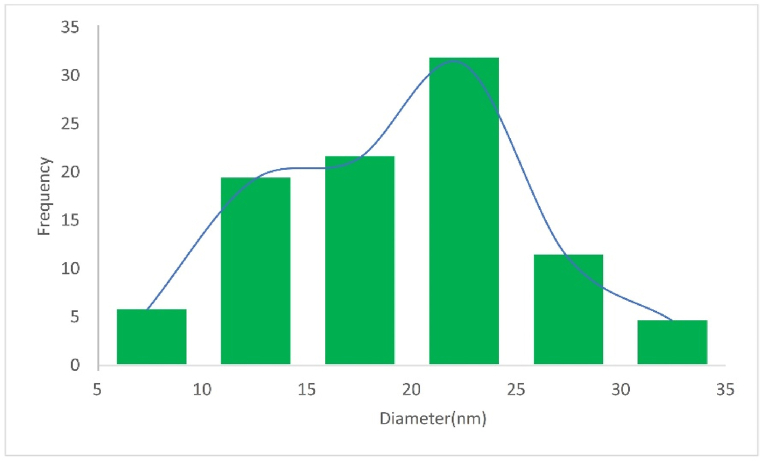
Particle size distribution histogram of ZrFe_2_O_4_@SiO_2_-S-CCH-Pd

Fig. 6 illustrates the particle size distribution of these catalysts, depicting the histogram of particle sizes extracted from SEM images. It is evident from the histogram that the ZrFe_2_O_4_@SiO_2_-S-CCH-Pd catalyst exhibits uniform diameters in the SEM images.

**Fig. 7:**
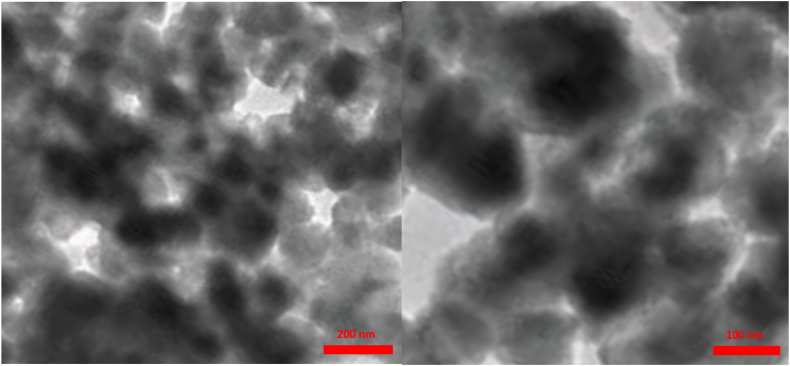
TEM images of ZrFe_2_O_4_@SiO_2_-S-CCH-Pd.

The structures and morphologies of the nanoparticles were characterized using transmission electron microscopy (TEM) (Fig. 7). As shown in Fig. 7, a basically core‐shell structure (dark colored core for ZrFe_2_O_4_@SiO_2_ nanoparticles and light‐colored shell for -S-CCH-Pd) was obtained (Fig. 7).

Then, with the help of ICP technique, the amount of Pd in the primary catalyst and the amounts of palladium leaching after recycling were checked. Based on the obtained results, the amounts of palladium in fresh and reused nanocatalysts are 1.8 × )10 (^−4^ and 1.6 × )10 (^−4^ mol. g^−1^ respectively, which shows that palladium leaching from the ZrFe_2_O_4_@SiO_2_-S-CCH-Pd framework is low.

**Fig. 8:**
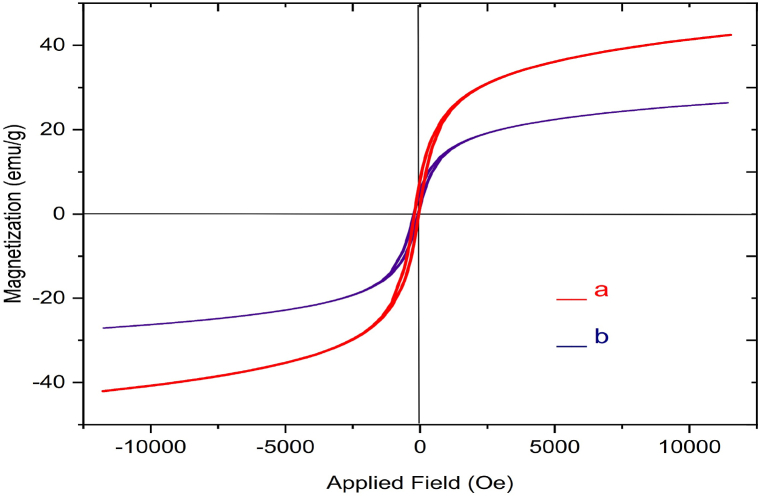
VSM curves of (a) ZrFe_2_O_4_ (b) ZrFe_2_O_4_@SiO_2_-S-CCH-Pd.

To investigate the magnetization of a) ZrFe_2_O_4_, and (b) ZrFe_2_O_4_@SiO_2_-S-CCH-Pd VSM was employed at room temperature (Fig. 8). As it could be perceived from the resulting magnetization curves, the saturation magnetization amount of the a) ZrFe_2_O_4_, and (b) ZrFe_2_O_4_@SiO_2_-S-CCH-Pd were 42 and 27 emu. g^−1^ respectively. The decrease of saturation magnetization in the prepared catalyst is due to the stabilization of the CCH-Pd complex on the ZrFe_2_O_4_ surface.

### 2.5 Catalytic study

In the next step, in order to optimize the reaction conditions, the catalytic activity of the ZrFe_2_O_4_@SiO_2_-S-CCH-Pd nanocomposite was investigated in the Suzuki-like cross-coupling reaction. The coupling reaction of iodobenzene and phenylboronic acid was chosen as a model reaction. Next, the influence of various experimental parameters such as solvent, catalyst loading and base and reaction temperature on the model reaction was checked (Table 1). From the obtained results, it can be concluded that the optimal conditions for this reaction are ZrFe_2_O_4_@SiO_2_-S-CCH-Pd (0.02 g), K_2_CO_3_ (1.5 mmol) as the base in the H_2_O at 90 ^°^C.

**Table 1:** Optimization of the model reaction of iodobenzene with phenylboronic acid catalyzed by ZrFe_2_O_4_@SiO_2_-S-CCH-Pd.


Entry	Catalyst (g)	Solvent	Base	Temperature (°C)	Time (min)	Yield (%)^a^
1	–	H_2_O	K_2_CO_3_	90	1 days	NR
2	0.005	H_2_O	K_2_CO_3_	90	15	36
3	0.008	H_2_O	K_2_CO_3_	90	15	55
4	0.01	H_2_O	K_2_CO_3_	90	15	81
5	0.015	H_2_O	K_2_CO_3_	90	15	95
6	0.02	H_2_O	K_2_CO_3_	90	15	98
7	0.03	H_2_O	K_2_CO_3_	90	15	98
8	0.02	PEG-400	K_2_CO_3_	120	15	73
9	0.02	EtOH	K_2_CO_3_	Reflux	15	80
10	0.02	DMSO	K_2_CO_3_	Reflux	15	92
11	0.02	H_2_O	NaOH	90	15	90
12	0.02	H_2_O	Na_2_CO_3_	90	15	92
13	0.02	H_2_O	KOH	90	15	36
14	0.02	H_2_O	Et_3_N	90	15	30

After identifying the optimal conditions for the Suzuki reaction catalyzed by ZrFe_2_O_4_@SiO_2_-S-CCH-Pd, the activity of a wide range of aryl iodides and aryl bromides is evaluated and the results are summarized in Table 2. All reactions proceeded with high efficiency and without producing appreciable amounts of byproducts arising. In particular, the effective coupling of aryl chlorides, aryl iodides, and aryl bromides, containing para, meta, and ortho functionalities showed superior activity of the catalyst. Under these optimal conditions, the usefulness of ZrFe_2_O_4_@SiO_2_-S-CCH-Pd in the C-C coupling reaction involving the coupling of phenylboronic acid with aryl halide was investigated. The results obtained are given in Table 2.

**Table 2:**
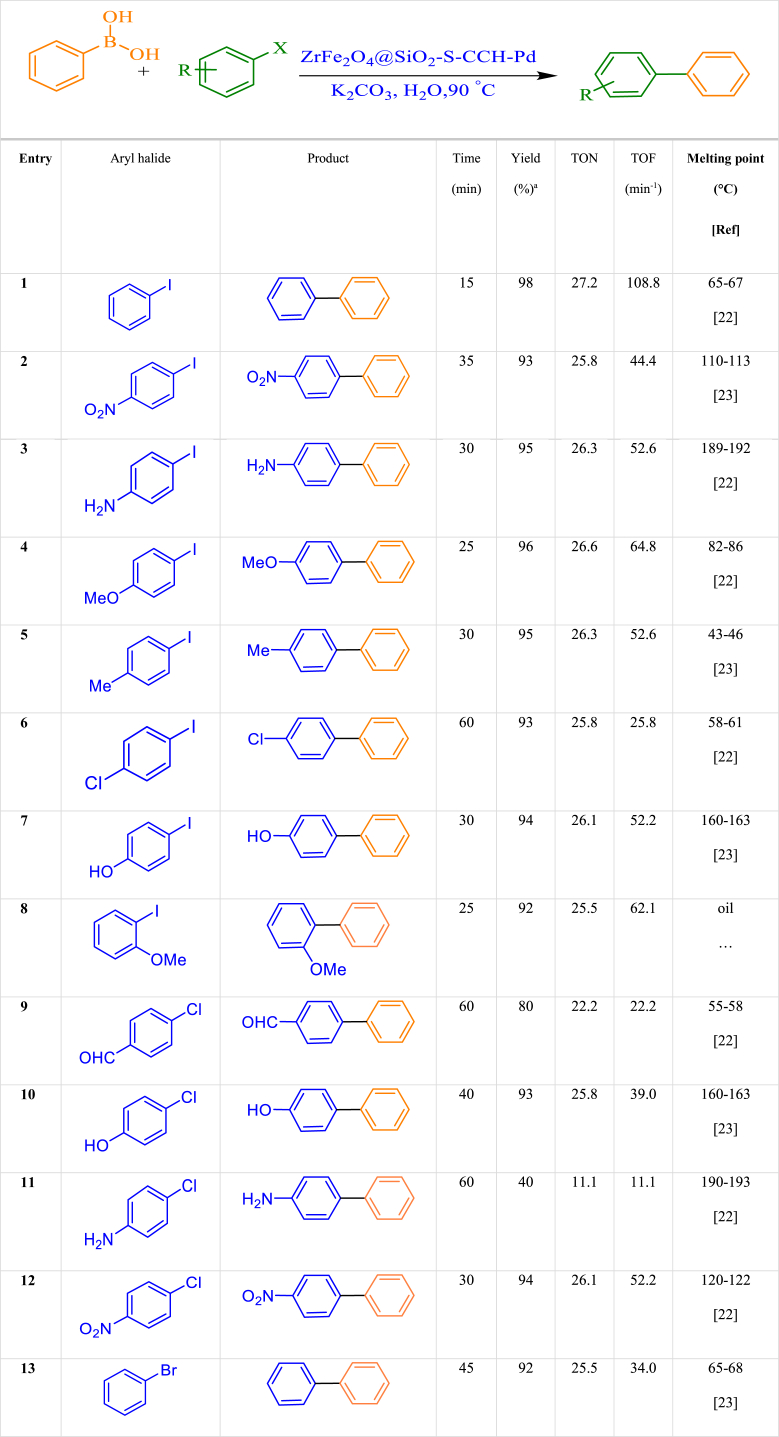

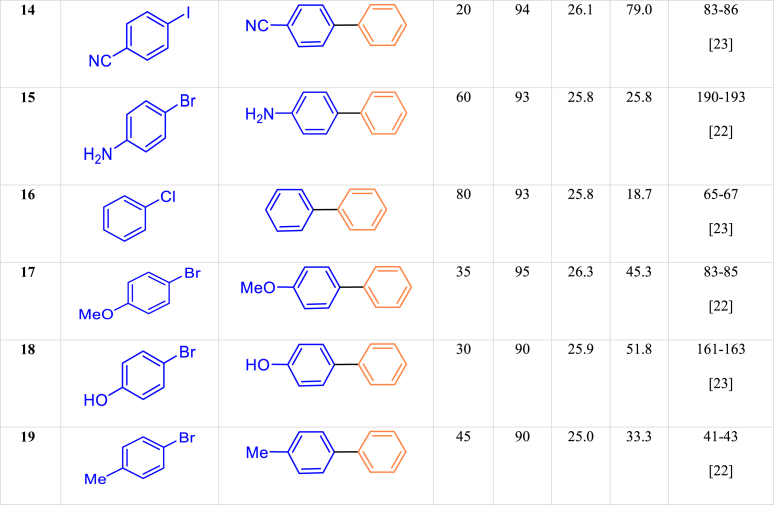
Synthesis of Suzuki reaction from aryl halides using ZrFe_2_O_4_@SiO_2_-S-CCH-Pd.

**Scheme 4:**
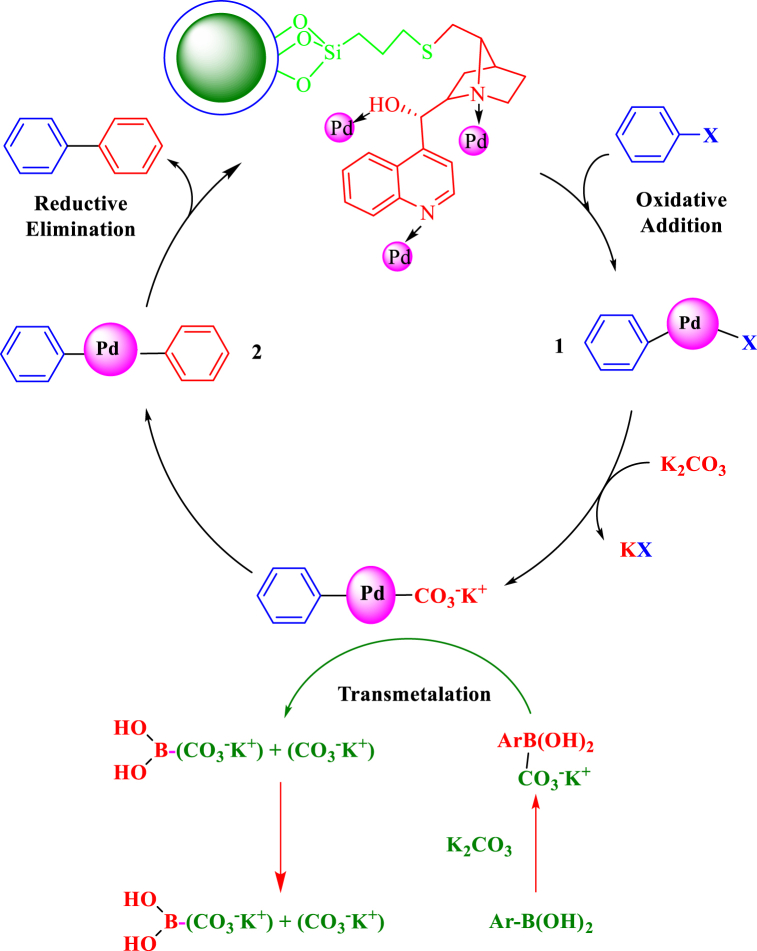
Possible mechanism for Suzuki reaction.

Although it is not possible to predict the course of the reaction clearly and completely here we proposed a mechanism for the Suzuki reaction. In the Suzuki coupling mechanism, aryl halide reacts with Pd through an additive-oxidation reaction. Following that, the base used in the reaction, which is potassium carbonate, activates it through phenylboronic acid and forms an ester derivative, phenyl boronate. Then, medium (2) is produced from the displacement reaction of the metal of medium (1) with the activated borane group. The final step in this reaction is an elimination-reduction step that produces the desired product and recovers the initial catalyst (Scheme 4).

After the synthesis and rigorous characterization of the catalysts, their application was important; thus, we started with the optimization of reaction conditions for both Figures. At the outset, we chose the Hiyama reaction of iodobenzene and triethoxyphenylsilane and a base as additives and ZrFe_2_O_4_@SiO_2_-S-CCH-Pd as a catalyst. We went through the investigations regarding the standardization of reaction conditions by varying different factors like solvent, bases, temperature and catalyst load in a model reaction of iodobenzene and triethoxyphenylsilane. The consequences are represented in Table 3. A screening of solvents was carried out with a series of solvents in the presence of K_2_CO_3_ as a base and 0.01 g of catalyst. There was no reaction output in PEG, EtOH, and H_2_O; however, the reaction responded excellently in a mixture of DMF and K_2_CO_3_. Moreover, the best result was achieved at 100 °C with 70 mg of catalyst. There is a definite role of the base in the reaction mechanism. We screened different bases like KOH, NaOH, and Na_2_CO_3_ under stabilized solvent, temperature, and catalyst conditions where K_2_CO_3_ showed the best productivity (Table 3).

**Table 3:** Optimization of the model reaction of triethoxyphenylsilane with iodobenzene catalyzed by ZrFe_2_O_4_@SiO_2_-S-CCH-Pd.


Entry	Catalyst (g)	Solvent	Base	Temperature (°C)	Time (min)	Yield (%)^a^
1	–	DMF	K_2_CO_3_	100	12 h	NR
2	0.005	DMF	K_2_CO_3_	100	40	46
3	0.008	DMF	K_2_CO_3_	100	40	79
4	0.01	DMF	K_2_CO_3_	100	40	97
5	0.02	DMF	K_2_CO_3_	100	40	97
6	0.01	PEG-400	K_2_CO_3_	120	40	69
7	0.01	H_2_O	K_2_CO_3_	Reflux	40	85
8	0.01	EtOH	K_2_CO_3_	Reflux	40	91
9	0.01	DMF	NaOH	100	40	90
10	0.01	DMF	KOH	100	40	89
11	0.01	DMF	Na_2_CO_3_	100	40	42

After optimizing the reaction conditions, the scope of the reaction was investigated by applying the supported ZrFe_2_O_4_@SiO_2_-S-CCH-Pd nanocatalyst to the Hiyama reaction of triethoxyphenylsilane and aryl halides. The results are listed in Table 4. As seen, excellent yields and TOFs were obtained for the examined aryl bromides and iodides (Table 4).

**Table 4:**
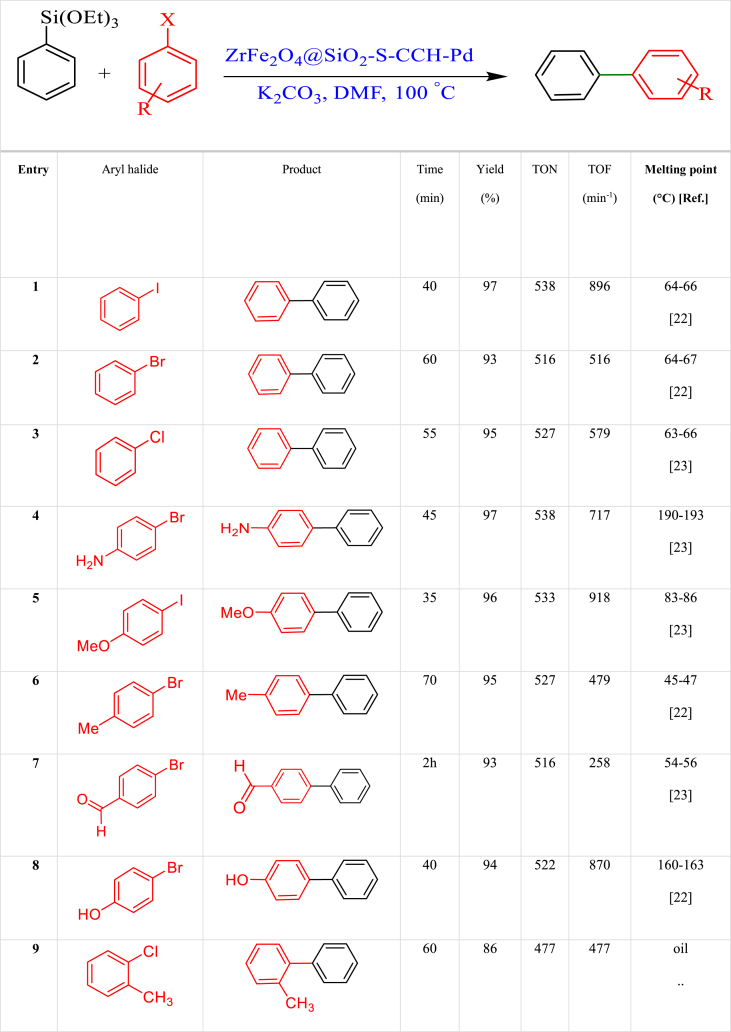
Synthesis of Hiyama reaction from aryl halides using ZrFe_2_O_4_@SiO_2_-S-CCH-Pd.

**Scheme 5:**
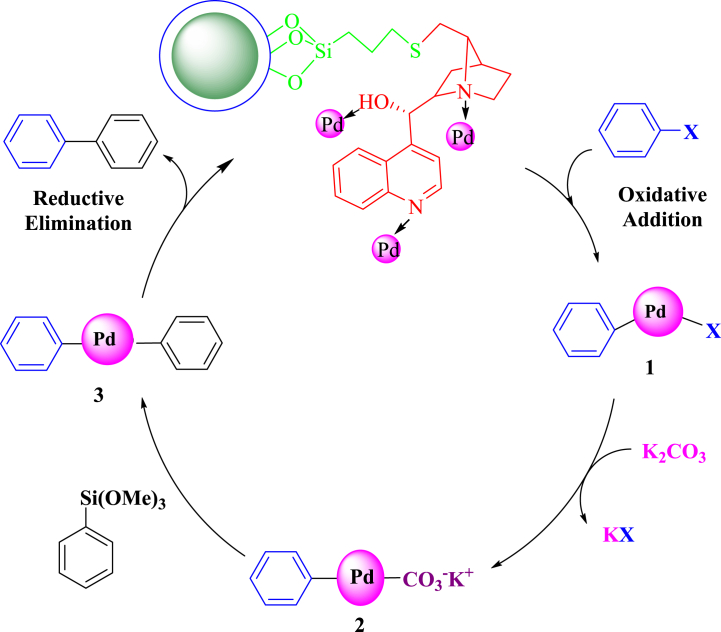
Proposed mechanism for Hiyama reaction.

The possible mechanism for the Hiyama reaction is shown in Scheme 5. First, oxidative addition of the aryl halide to Pd leads to the formation of complex 1. Complex 1 undergoes transmetalation wherein the nucleophile is transferred to Pd to produce intermediate complex 2. The reductive elimination of 6 gives the coupled product (complex 2) and regenerates Pd for the next catalytic cycle (Scheme 5).

## 3 Catalyst recyclability

**Fig. 9:**
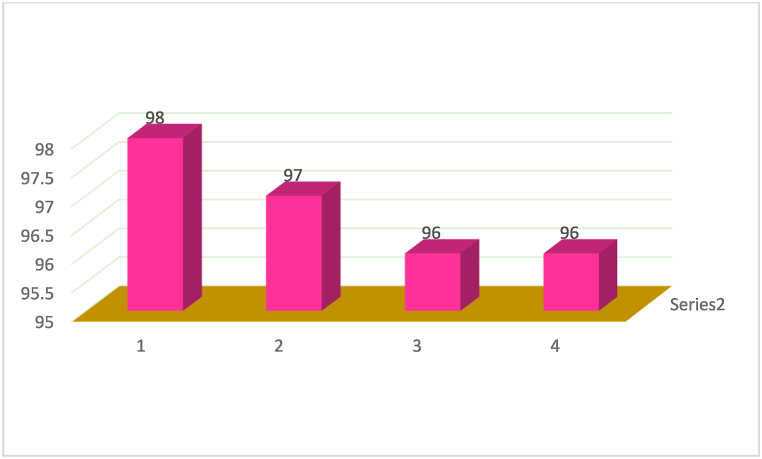
Recyclability of ZrFe_2_O_4_@SiO_2_-S-CCH-Pd in the synthesis of Suzuki reaction.

Next, to investigate the activity constancy of the catalyst, the iodobenzene with phenylboronic acid was examined as a model reaction in ethanol using 0.02 g of ZrFe_2_O_4_@SiO_2_-S-CCH-Pd. The as-prepared catalyst was easily recovered by a magnet as described in the experimental section. The ZrFe_2_O_4_@SiO_2_-S-CCH-Pd was reused in four cycles without loss of catalytic activity (Fig. 9).

In the next step, a contrast of the behavior of multiple catalysts with ZrFe_2_O_4_@SiO_2_-S-CCH-Pd in the Suzuki reaction released in the literature is recorded in Table 5. As shown in Table 5, ZrFe_2_O_4_@SiO_2_-S-CCH-Pd catalyst shows higher efficiency compared to other listed catalysts.

**Table 5:** Comparing catalytic activity of ZrFe_2_O_4_@SiO_2_-S-CCH-Pd with previously reported methods in the Suzuki reaction.

Entry	Ar-X	Catalyst	Time (mine)	Yield (%)	Ref.
1		Pd@DCA-MCM	120	94	[22]
2		VO (IV)–MCM-41	720	100	[23]
3		MCM-Pd	1440	74	[24]
4		Pd (0)- Schif-base@MCM-41	70	95	[25]
5		ZrFe_2_O_4_@SiO_2_-S-CCH-Pd	15	98	This work

## 4 Conclusion

In summary, we have successfully synthesized an effective and facile procedure for the synthesis of ZrFe_2_O_4_@SiO_2_-S-CCH-Pd as a magnetically recoverable catalyst with characterization by a variety of techniques. The structure, morphology, and physicochemical properties of the particles were characterized through different analytical techniques, including fourier transformed infrared spectroscopy (FT-IR), scanning electron microscopy (SEM), transmission electron microscopy (TEM), inductively coupled plasma (ICP), X-ray powder diffraction (XRD), thermogravimetric analysis (TGA), energy dispersive X-ray spectroscopy (EDS) and vibrating sample magnetometer (VSM) techniques. Moreover, the catalytic activity of this nanocomposite was investigated in Suzuki and Hiyama coupling reactions of aryl halides with phenylboronic acid as phenylating source and Triethoxyphenylsilane in H_2_O and DMF under green conditions. Also, high surface area, convenient recoverability and reusability for several times without any significant loss of catalyst activity, eco-friendly procedure, operational simplicity, cheap and chemically stable reagents, ease of separation by simple filtration, the use of commercially available materials, good reaction times, simple practical methodology and ease of use make the prepared catalyst a promising candidate for potential applications in some organic reactions.

## Data availability

All data generated or analyzed during this study are included in this published article and its supplementary information files.

## CRediT authorship contribution statement

**Zhino Mohammed Sdiq:** Investigation, Writing – review & editing. **Hadi Pourmokhtar:** Writing – review & editing, Writing – original draft, Visualization, Software, Project administration, Investigation, Funding acquisition. **Fatemeh Keshavarzi:** Funding acquisition, Investigation, Writing – review & editing.

## Declaration of competing interest

The authors declare that they have no known competing financial interests or personal relationships that could have appeared to influence the work reported in this paper.
