# Supplementary material for: CCH-Pd complex anchored on ZrFe2O4 nanoparticles as a novel magnetic catalytic for C-C coupling reactions
Source: Heliyon. 2024 Sep 19;10(18):e37683. doi: 10.1016/j.heliyon.2024.e37683 (PMC11447329; doi:10.1016/j.heliyon.2024.e37683)
Supplement: Multimedia component 1 [file mmc1.docx]

**CCH-Pd complex anchored on ZrFe_2_O_4_ nanoparticles as a novel magnetic catalytic for C-C coupling reactions**

Zhino Mohammed Sdiq^1^, Hadi Pourmokhtar *****^2^, Fatemeh Keshavarzi^3^

**^1^** bright technical and vocational institute Sulaymaniyah, Iraq. Email: [zhinomohammedsdiq@gmail.com](mailto:zhinomohammedsdiq@gmail.com)

^2^ Faculty of chemistry, university of Tabriz, Iran

^3^ Student Research Committe, School of Public Health, Kermanshah University of Medical Sciences, Kermanshah, Iran


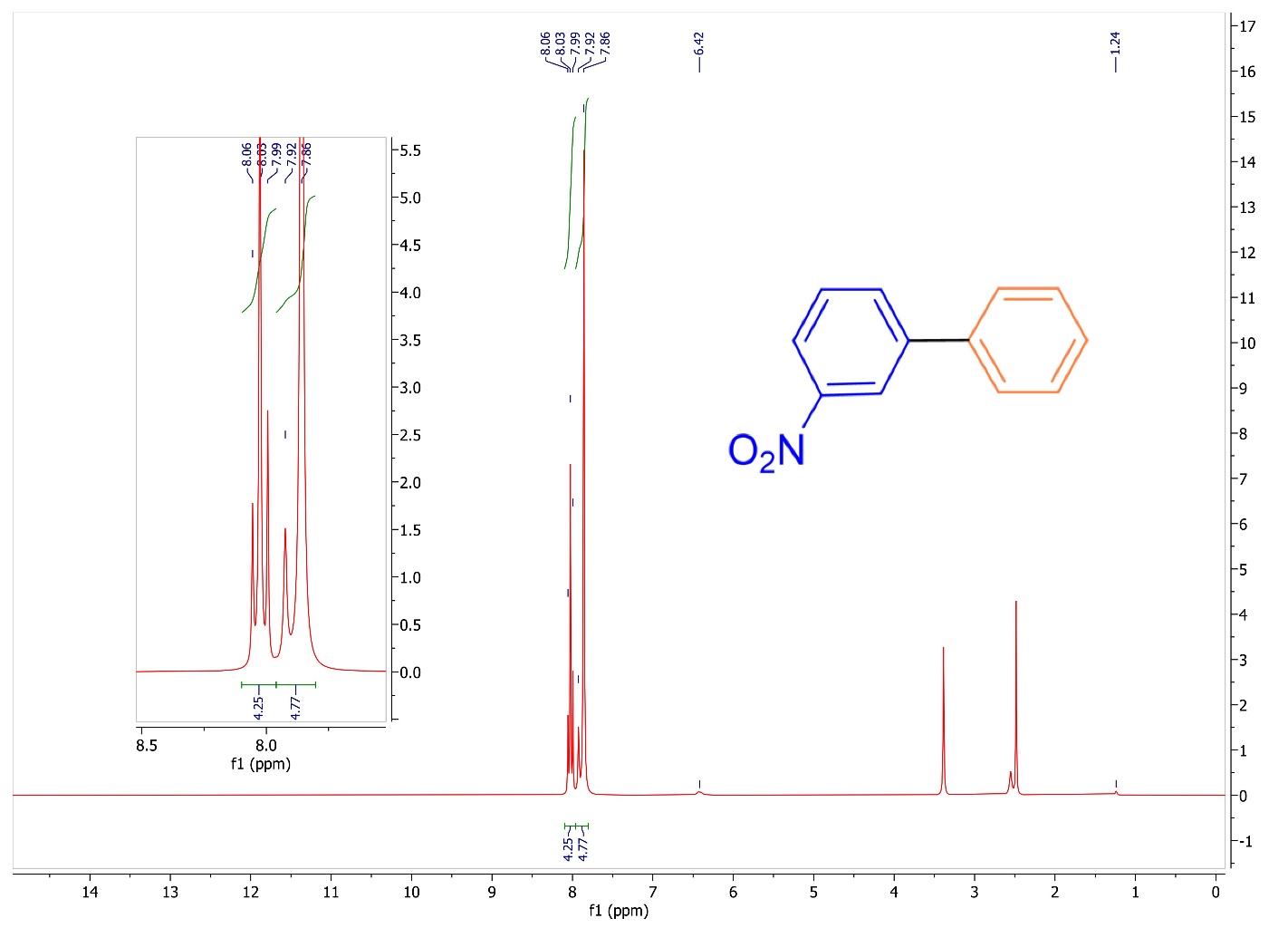


**S_1_)** **3-nitro-1,1'-biphenyl**


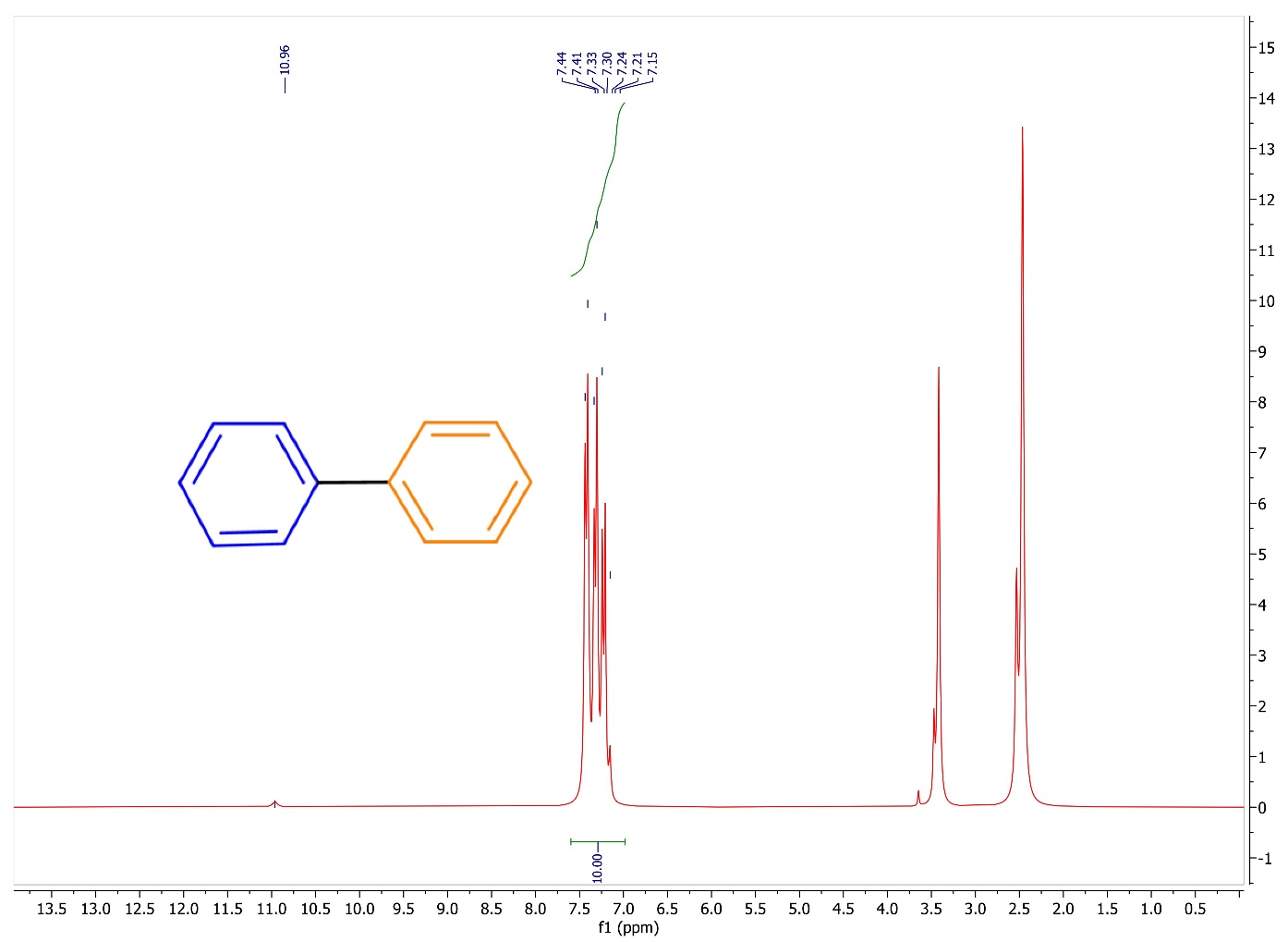


**S_2_)** **1,1'-biphenyl**


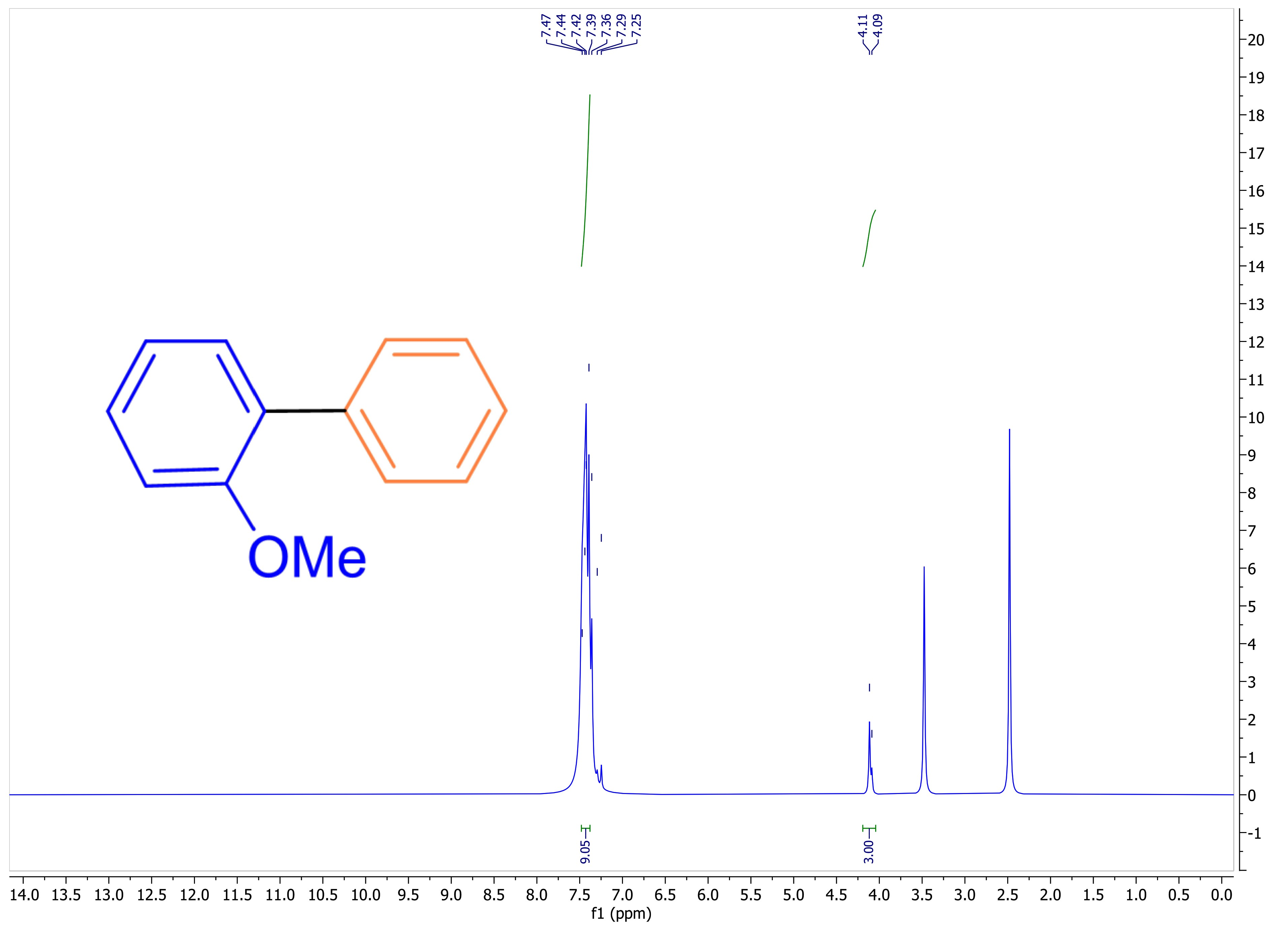


**S_3_) 2-MeO-1,1'-biphenyl**


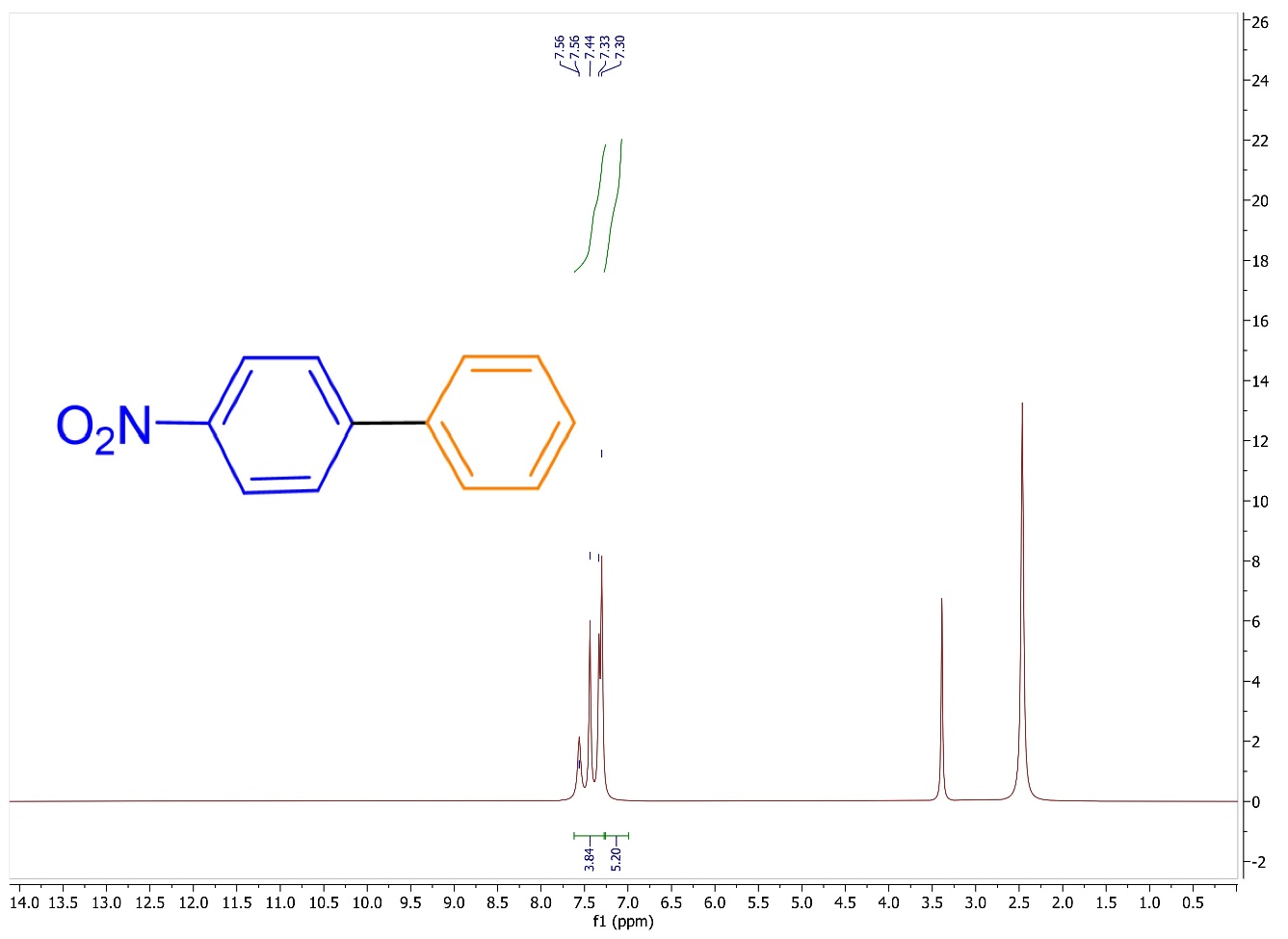


**S_4_) 4-nitro-1,1'-biphenyl**


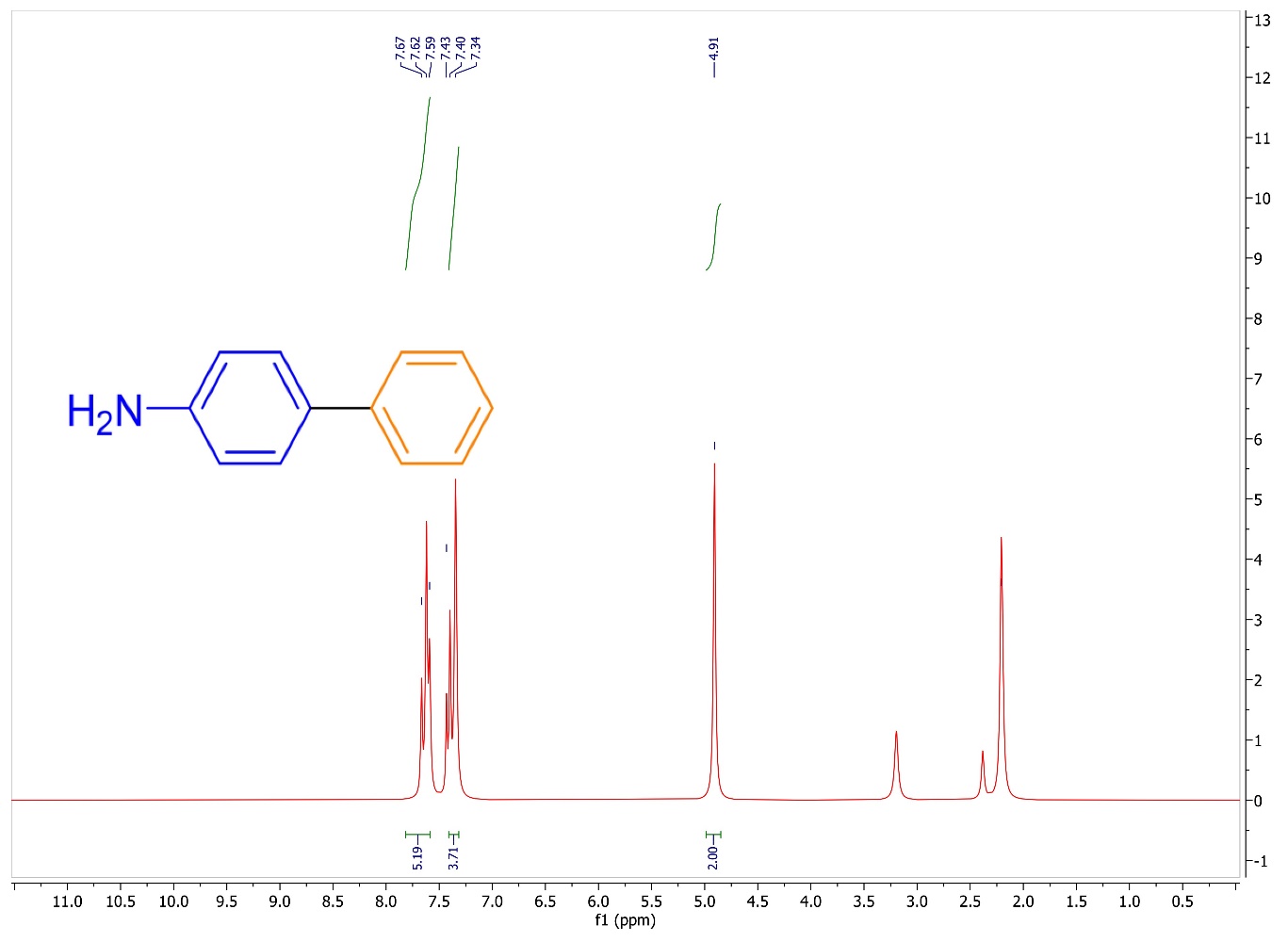


**S_5_) [1,1'-biphenyl]-4-amine**
